# R103 and R115 Affinity Mutants of ATeam ATP Biosensors

**DOI:** 10.3390/s25196180

**Published:** 2025-10-06

**Authors:** Autumn Cholger, Jason M. Conley, Stephen A. Valentino, Elaine Colomb, Olivia de Cuba, Jacob Kress, Mathew Tantama

**Affiliations:** 1 Department of Chemistry, Purdue University, West Lafayette, IN 47907, USA; 2 Department of Chemistry & Biochemistry Program, Wellesley College, Wellesley, MA 02481, USA

**Keywords:** ATP biosensor, affinity mutant, FRET

## Abstract

Adenosine triphosphate (ATP) varies from nanomolar to millimolar levels across the physiological landscapes in which it serves as an energy carrier, phosphate donor, and purinergic signaling molecule. To measure these vastly different concentrations, genetically encoded sensors with different affinities are needed to match the particular ATP range and application. To this end, we mutagenized two key arginine residues in the ATP-binding domain of the ATeam family of sensors to explore how charge neutralization and charge reversal affect ATP affinity. As a result, we generated an extended family of affinity mutants with apparent dissociation constants ranging from sub-micromolar to millimolar. We then carried out live-cell imaging to demonstrate the utility of different affinity mutants in detecting mild versus extreme metabolic inhibition. Overall, these sensors add to the toolbox for understanding ATP dynamics in and around cells.

## 1. Introduction

Tissue and cellular concentrations of ATP span several orders of magnitude from hundreds of nanomolar in the extracellular space to hundreds of millimolar in secretory vesicles [1,2,3]. To study cytosolic and mitochondrial ATP dynamics in the low millimolar range, Imamura and co-workers developed the ATeam family of sensors that have been used extensively [1]. The ATeam sensors are ratiometric, using the Förster resonance energy transfer (FRET) pair mseCFP and mVenus flanking an ATP-binding domain. The ATP-binding domain consists of an ATP-binding ε regulatory subunit of a prokaryotic ATP synthase. In their original work, Imamura and co-workers hypothesized that sensors with different ATP affinities could be generated using regulatory subunits from different species or by making mutations in the ATP-binding domain that would alter the ATP-induced conformational dynamics. They demonstrated this beautifully by optimizing the ATeam1.03 and ATeam3.10 sensors with affinity variants. This family of sensors and its relatives use the ATP synthase ε subunit from *Bacillus subtilis* [4] or *Bacillus* sp. PS3 [5,6] as low-affinity or high-affinity ATP-binding domains, respectively [1,7,8,9,10,11,12,13,14,15]. Subsequently, several others have focused on testing different mutations of these ε subunits, and mutations in the N-terminal β-sandwich and C-terminal α-helical domains have been made to further tune the affinity for ATP [1,8,13,14,16]. For example, Lobas and co-workers made T9V, N78Y, A95K, and A119S mutations in the *B.* sp. PS3 ε subunit to generate iATPSnFR [13], and later Marvin and co-workers further varied the 95 and 119 positions to generate the iATPSnFR2 affinity variants [14]. In order to engineer a sensor optimized for studies at 25 °C instead of 37 °C, Tsuyama and Kishikawa and co-workers explored the interface of the N-terminal and C-terminal domains [8]. They identified the M60N and K132L mutations in the *B. subtilis* ε subunit to lower the affinity of the ATeam1.03NL variant. Interestingly, the iATPSnFR2.A95A.A119L variant was shown to have good temperature independence [14], but the ATeam1.03NL sensor has been the primary example focused on optimization for work at room temperature [8]. Given the importance of non-mammalian model organisms, such as worms and flies, as well as many cell culture experiments that are carried out at lower temperatures, we sought to develop new affinity variants of the ATeam family that expand the ATP-sensing range at room temperature.

**Figure 1:**
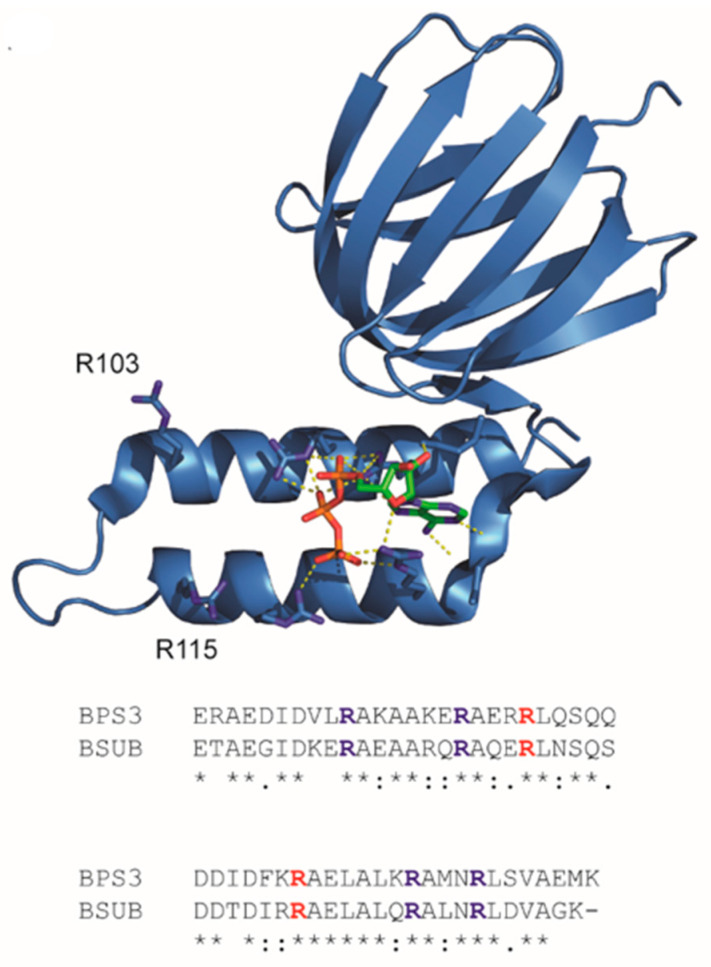
Structure of the *Bacillus* sp. PS3 ATP synthase ε subunit. Top: X-ray crystal structure, PDB: 2E5Y [20]. Bottom: Sequence alignment of the C-terminal ATP-binding domains for the *Bacillus* sp. PS3 and *Bacillus subtilis* ε subunits: conserved residues R92, R99, R122, and R126 that directly interact with ATP are shown in blue; conserved residues R103 and R115 mutagenized in this study are shown in red.

To this end, Kato-Yamada reported that the R103A/R115A double mutation in the *Bacillus* sp. PS3 ε subunit confers greatly increased ATP affinity with an apparent dissociation constant two orders of magnitude lower than the wildtype [17]. Unlike arginine residues R92, R99, R122, and R126 that directly interact with ATP, residues R103 and R115 do not interact (Figure 1). Rather, Krah and co-workers proposed that residues R103 and R115 influence the hydrogen bonding network and electrostatics of the adjacent ATP-binding site interactions [18,19]. These arginine residues are conserved in the *Bacillus subtilis* ε subunit, and therefore, we asked whether these residues could be varied to further tune the ATP affinity of the ATeam sensors. We explored combinations of single and double mutants with charge neutralization and charge reversal, and as a result, we characterized a family of ATeam mutants tuned to sub-micromolar to millimolar ATP affinities.

## 2. Materials and Methods

***Molecular Biology.*** Affinity mutants were generated with the NEB Q5 Site-Directed Mutagenesis kit (NEB, Ipswich, MA, USA) using the parent plasmids pRsetB-ATeam1.03, pRsetB-ATeam1.03YEMK, and pRsetB-ATeam3.10 from Imamura [1]. Briefly, overlapping mutagenic primers were designed according to the manufacturer’s instructions. Double mutants were generated by carrying out a second round of mutagenesis on the single mutants as templates using the same mutagenic primers. All mutants were verified by Sanger sequencing.

***Protein Characterization.*** Polyhistidine-tagged proteins were expressed in BL21(DE3) *E. coli* (NEB, Ipswich, MA, USA) in Auto Induction Media (Formedium, Norfolk, UK) and purified by nickel-affinity (GE Chelating Sepharose, Cytiva, Marlborough, MA, USA) fast protein liquid chromatography (AKTA, Cytiva, Marlborough, MA, USA). Assays were carried out at room temperature with 0.125 µM protein in assay buffer (50 mM MOPS-KOH, pH 7.3, 50 mM KCl, 0.5 mM MgCl_2_, 0.05% Triton-X100). ATP stocks were neutralized to pH 7, and a total MgCl_2_ concentration was added to maintain 0.5 mM free Mg^2+^ [21]. Samples were prepared in clear-bottom, black 96-well plates (Greiner Bio-One, Monroe, NC, USA). Sensor protein and ATP at a concentration dictated by each point on the dose–response curve were added to a single well with assay buffer and allowed to equilibrate. Samples were measured with n = 4 to 9 replicates. Steady-state fluorescence was measured on a BioTek Synergy H4 using 420/50 nm excitation and 485/20 nm or 528/20 nm emission bandpass filters (BioTek, Winooski, VT, USA). Donor fluorescence lifetimes were measured using an Edinburgh Instruments FS5+ time-resolved fluorometer with an NKT-Fianium WhiteLaseMicro supercontinuum pulsed laser (Edinburgh Instruments, Livingston, UK). Donor fluorescence lifetime distribution analysis was carried out using the MemExp software [22,23,24].

***Microscopy.*** HEK293 cells were cultured in low-glucose Dulbecco’s modified Eagle’s medium supplemented with 10% cosmic calf serum (Gibco, Thermo Fisher Scientific, Waltham, MA, USA). For imaging studies, cells were plated on 18 mm #1.5 coverslips and transfected with Effectene (Qiagen, Germantown, MD, USA) or by the calcium phosphate method. Prior to imaging, cells were equilibrated for at least 20 min at room temperature in imaging solution (15 mM HEPES, pH 7.3, 1.25 mM NaH_2_PO_4_, 130 mM NaCl, 3 mM KCl, 2 mM CaCl_2_, 1 mM MgCl_2_, 3 mM NaHCO_3_) supplemented with a glucose concentration dependent on the specific experiment. Live-cell microscopy was carried out at room temperature on an Olympus IX83 inverted fluorescence microscope with a 20X/0.75 NA air objective illuminated by a Lumencor SpectraX light engine and imaged with an Andor Zyla 4.2 sCMOS camera. An ECFP/EYFP/mCherry multiband beamsplitter (Chroma 69008bs) was used with bandpass excitation and emission filters for each channel: cyan 438/29 nm and 470/24 nm; yellow 510/10 nm and 540/30 nm; sensitized FRET 438/29 nm and 540/30 nm; red 575/25 nm and 631/28 nm. Andor iQ3 software was used for image acquisition, and ImageJ/FIJI was used for image analysis. (Evident Scientific, Waltham, MA, USA) Cell masks were created using a threshold of two times the background and applied to background-subtracted images. Pixel-by-pixel ratio images were created from sensitized FRET and donor cyan channels, and regions of interest were manually drawn. OriginPro2017 was used for curve fitting.

## 3. Results and Discussion

### 3.1. Tuning a High-Affinity Family of ATeam3.10 Sensors

The ATeam3.10 sensor utilizes the higher-affinity *Bacillus* sp. PS3 ε subunit. Using Cy3-labeled protein, Kato-Yamada demonstrated that the R103A/R115A double mutation increases ATP affinity from 4.3 µM for the wildtype [16] to 52 nM for the double mutant [17]. Subsequently, Krah and co-workers carried out a molecular dynamics study to predict whether the individual R103A and R115A mutations would also increase ATP affinity on their own [18,19]. Mutations of these two residues have not previously been explored in detail for the ATeam family of sensors, so we carried out a mutational analysis of these two arginines to determine if they can fine-tune the ATP affinity of ATeam3.10.

**Figure 2:**
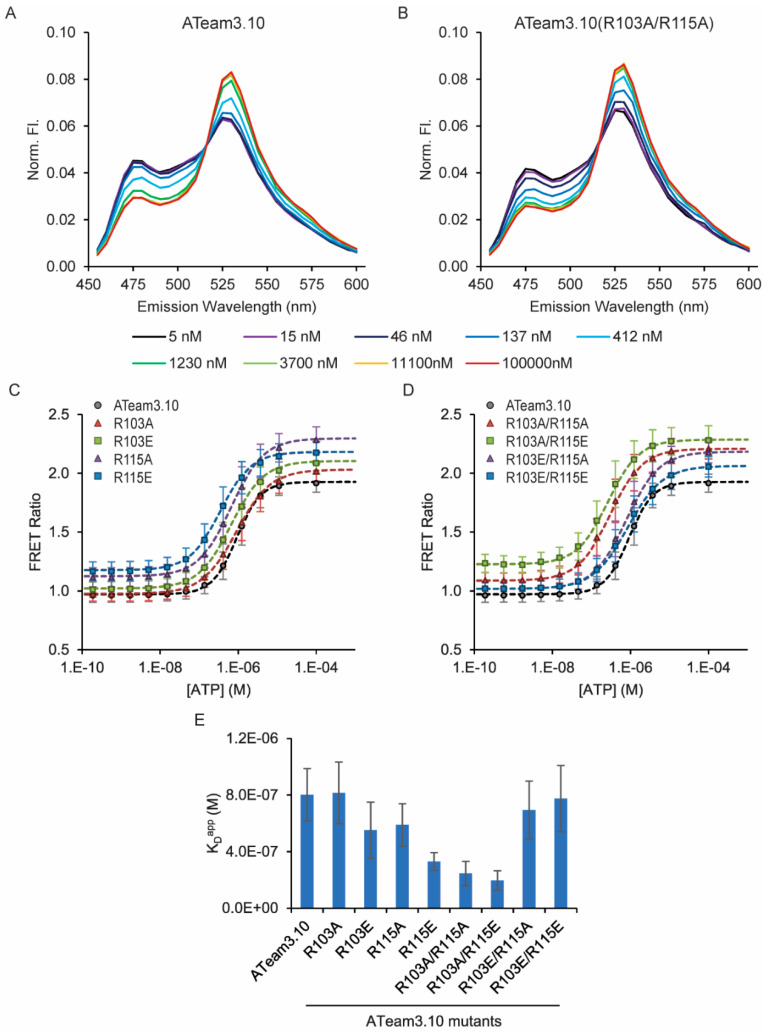
ATeam3.10 R103 and R115 single and double mutants tune ATP affinity. ATP dose responses were measured using purified protein in solution. FRET ratios were calculated as sensitized acceptor emission-to-donor emission ratios. (**A**) Example fluorescence emission spectra for (**A**) wildtype ATeam3.10 and (**B**) the R103A/R115A double mutant. ATP dose response curves for (**C**) single and (**D**) double mutants. Apparent dissociation constants are summarized in (**E**).

We measured the ATP dose–response curves for all combinations of single and double mutants consisting of R-to-A charge neutralization or R-to-E charge reversal (Table 1, Figure 2). We first confirmed that the R103A/R115A confers a significantly increased affinity from a K_D_^app^ of 800 ± 300 nM (n = 9) for the wildtype ATeam3.10 down to a K_D_^app^ of 200 ± 100 nM (n = 4) (mean ± standard deviation, *p* = 0.004, 2-tailed *t*-test). We observed a 4-fold increase in affinity, whereas Kato-Yamada observed an 83-fold increase in affinity. The difference between our observations likely reflects the sensitivity of the ε subunit to the label used [6]. Kato-Yamada used a relatively small Cy3 label with a cysteine mutant (Q107C) of the ε subunit, whereas the ε subunit is fused to two large fluorescent proteins in ATeam3.10. Despite this difference, the increase in affinity could still be of particular use for developing an ATeam-based sensor to detect the low levels of extracellular ATP [2,3,11,13,25].

Next, we examined the single R103A and R115A mutants that Krah and co-workers predicted would exhibit increased ATP affinity [18,19]. However, we did not observe a statistically significant decrease in the apparent dissociation constant for either of the individual alanine mutants (Table 1, Figure 2). Instead, we found that charge reversal of the R115E single mutant, as well as the mixed double mutant R103A/R115E, both cause an increase in ATP affinity just like the R103A/R115A charge neutralization double mutant. In contrast, neither the single R103E charge reversal mutant nor the mixed R103E/R115A double mutant significantly increases ATP affinity. Given the differences between mutants, we carried out mutant cycle analysis [26], but the coupling energies are small and do not suggest strong interactions between the residues (Figure S1). However, there is clearly some interaction between sites. For example, whereas the R103A/R115E mixed double mutant has a higher affinity, charge reversal at both sites causes the R103E/R115E double mutant to have an ATP affinity similar to the wildtype ATeam3.10, potentially due to electrostatic repulsion. Notably, this R103E/R115E is the only mutant exhibiting an energetic penalty for ATP binding. All other mutations either increase ATP affinity or are tolerated with no significant change relative to the wildtype.

**Figure 3:**
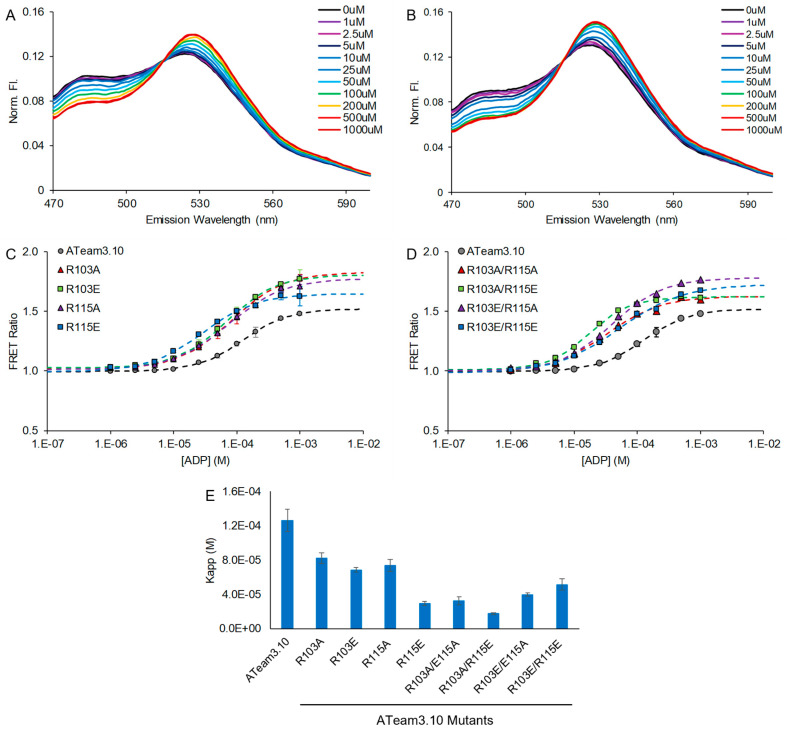
ATeam3.10 R103 and R115 single and double mutants also increase ADP affinity. ADP dose responses were measured using purified protein in solution. FRET ratios were calculated as sensitized acceptor emission-to-donor emission ratios. (**A**) Example fluorescence emission spectra for (**A**) wildtype ATeam3.10 and (**B**) the R103A/R115A double mutant. ADP dose response curves for (**C**) single and (**D**) double mutants. Apparent dissociation constants are summarized in (**E**).

The original ATeam3.10 exhibits high selectivity for ATP binding over ADP binding [1], and so we also asked how the R103 and R115 single and double mutants affect apparent ADP affinity. First, we confirmed that the original ATeam3.10 has very high selectivity for ATP over ADP, with a >150-fold difference in apparent affinities (Table 2, Figure 3). For both single and double mutants, ADP affinity was increased, similar to the trend observed for the mutational effects on ATP affinity. One interesting difference is that the R103E/R115A and R103E/R115E double mutants show somewhat lower selectivity, though they both still exhibit >50-fold difference in apparent ADP and ATP affinities. It may be that the glutamate residue in these mutational backgrounds is positioned closer to the ADP phosphates to cause electrostatic repulsion. Despite this, overall, the R103 and R115 mutants of ATeam3.10 have excellent selectivity for ATP over ADP.

### 3.2. Tuning Affinities of ATeam1.03 and ATeam1.03YEMK

**Figure 4:**
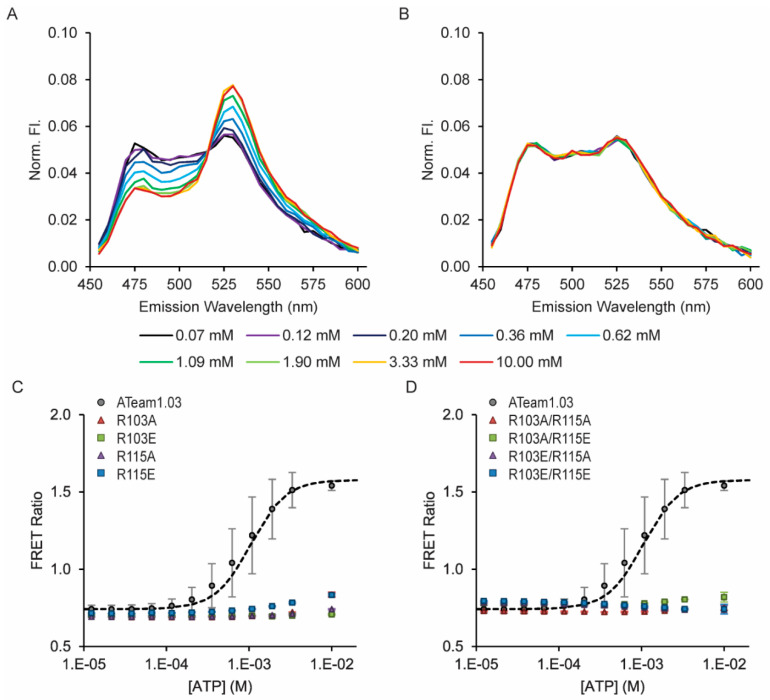
All ATeam1.03 R103 and R115 single and double mutants cause loss of ATP affinity. ATP dose responses were measured using purified protein in solution. FRET ratios were calculated as sensitized acceptor emission-to-donor emission ratios. (**A**) Example fluorescence emission spectra for (**A**) wildtype ATeam1.03 and (**B**) the non-binding R103A/R115A double mutant. ATP dose response curves for (**C**) single and (**D**) double mutants.

The ATeam1.03 sensor utilizes the lower-affinity *Bacillus subtilis* ε subunit, and the R103 and R115 residues are conserved in its C-terminal ATP-binding domain. Although there is no X-ray crystal structure available for the *Bacillus subtilis* ε subunit, I-TASSER and AlphaFold3 models predict that it is highly homologous to the *Bacillus* sp. PS3 ε subunit structure. Therefore, we tested whether mutagenesis at these positions could also tune the affinity of ATeam1.03. Surprisingly, all of the R-to-A or R-to-E single or double mutations cause a complete loss of ATP binding (Figure 4, Table 3) and ADP binding (Figure S2).

**Figure 5:**
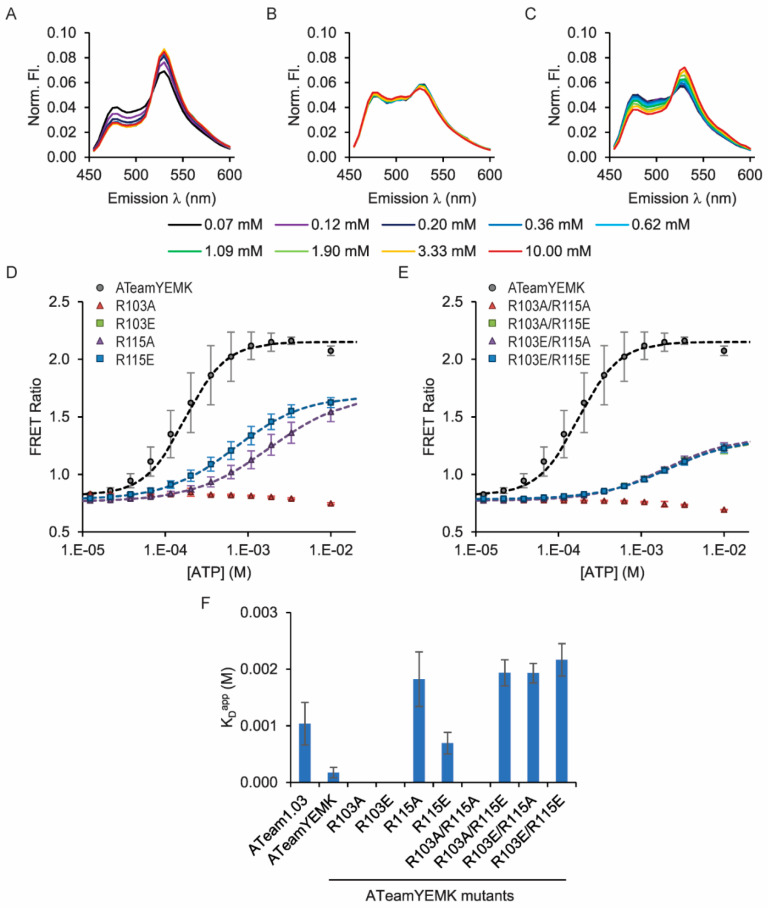
ATeam1.03YEMK R103 and R115 single and double mutants tune ATP affinity. ATP dose responses were measured using purified protein in solution. FRET ratios were calculated as sensitized acceptor emission-to-donor emission ratios. (**A**) Example fluorescence emission spectra for (**A**) wildtype ATeam1.03YEMK, (**B**) the R103A/R115A double mutant, and (**C**) the R103A/R115E double mutant. ATP dose response curves for (**D**) single and (**E**) double mutants. Apparent dissociation constants are summarized in (**F**).

Even though none of the mutations are tolerated in ATeam1.03, we decided to test the R103 and R115 single and double mutations in the ATeam1.03YEMK sensor because of the modifications already made to its ATP-binding domain. In this sensor, four residues at the putative interface between the N-terminal and C-terminal domains of the *Bacillus subtilis* ε subunit are exchanged to match the *Bacillus* sp. PS3 ε subunit, and these mutations cause an increased ATP affinity relative to ATeam1.03 [1]. In this ATeam1.03YEMK background, five of the eight R103 and R115 mutant combinations are tolerated, but in contrast to ATeam3.10, all of the mutations cause a significant decrease in ATP affinity relative to the wildtype ATeam1.03YEMK (Table 3, Figure 5) and loss of ADP binding (Figure S3). Notably, the R103A/R115A double mutant, which confers high affinity in the ATeam3.10 sensor, causes loss of ATP binding in both ATeam1.03 and ATeam1.03YEMK backgrounds. Interestingly, charge reversal at both sites causes the ATeam1.03YEMK(R103E/R115E) double mutant to exhibit an 11-fold decrease in affinity, which is advantageous to further expand the range of the affinity mutants characterized.

We also examined the Mg^2+^-dependence of ATP-binding. The *Bacillus* sp. PS3 ε subunit does not require Mg^2+^ to bind ATP, whereas the *Bacillus subtilis* ε subunit does require Mg^2+^ [1,4,5,16,20]. We confirmed that ATeam1.03 requires Mg^2+^ for ATP binding, but ATeam3.10 binds ATP independently of Mg^2+^ (Figure S4). Interestingly, we found that the wildtype ATeam1.03YEMK sensor can bind ATP in the absence of Mg^2+^, but that Mg^2+^ modifies its affinity (Figure S4). In contrast, the ATeam1.03YEMK(R115E) mutant binds ATP independently of Mg^2+^ (Figure S5). We also used time-resolved donor lifetime measurements to validate that the Mg^2+^ was not causing an artifact in acceptor fluorescence, which could be mistaken as a change in sensitized emission in steady-state measurements alone. Indeed, steady-state ratiometric and time-resolved donor lifetime measurements agree, demonstrating that the observations of Mg^2+^-dependence are truly changes in the FRET-based binding responses (Figures S4 and S5). Thus, the mutations also affect Mg^2+^ dependence in a complex fashion that should be taken into account for specific applications.

Given that all of the R103 and R115 mutations cause loss of function, we were curious if there was a change in the conformational dynamics of the ε subunit, possibly indicative of a larger-scale structural disruption. Yagi and co-workers previously demonstrated that the homologous *Bacillus* sp. PS3 ε subunit C-terminal domain adopts an extended flexible helix conformation in the absence of ATP [20]. We therefore asked if a non-binding mutation causes increased flexibility that would manifest as a broadening of basal FRET efficiencies. However, donor fluorescence lifetime distribution analysis does not support this [22,23,24]. For example, the ATeam1.03YEMK(R103A) single mutant does not exhibit any change in the mean or variance of donor fluorescence lifetime distribution upon the addition of ATP, and it is comparable to that of the ATeam1.03YEMK parent in the absence of ATP (Figure S6).

In a similar vein, we also asked whether the non-binding R103A mutation could somehow alter the ATP-induced conformational change so that the fluorescent proteins cannot achieve proper orientation for FRET. However, acceptor fluorescence polarization does not support this (Figure S7). In the wildtype ATeam1.03YEMK, ATP-binding causes a conformational change in the ε subunit, bringing the mseCFP and mVenus FRET pair together. This conformational change causes a compaction of the sensor “particle” and should decrease the rotational correlation time. Indeed, the addition of ATP to the ATeam1.03YEMK wildtype causes an increase in the mVenus acceptor fluorescence polarization, indicating slower rotation due to a larger effective particle size (Figure S7). In contrast, the R103A mutant exhibits no change in response to ATP addition, and its acceptor fluorescence polarization is comparable to that of parent ATeam1.03YEMK in the absence of ATP. Thus, the loss-of-function mutations appear to primarily affect ATP binding and do not drastically disrupt the structure of the ε subunit or function of the FRET pair.

### 3.3. Imaging Metabolic Dynamics

Finally, we demonstrated proof of concept that affinity mutants can differentially respond to varied levels of metabolic stress in live-cell imaging experiments. To do this, we treated HEK293 cells with the glycolytic inhibitor 2-deoxyglucose (DG), the combination of mitochondrial inhibitors trifluoromethoxy carbonylcyanide phenylhydrazone (FCCP) plus oligomycin, or all three in the presence or absence of glucose. In order to directly compare responses, we co-transfected one set of cells with the ATeam1.03YEMK sensor with excess nuclear-targeted H2B-mApple as a marker for identification. Separately, we transfected another set of cells with the ATeam1.03YEMK(R115E) sensor only. Cells were then mixed so they could be imaged simultaneously.

We chose to compare the R115E mutant to the wildtype ATeam1.03YEMK sensor because it represented a more challenging distinction than with one of the even lower-affinity mutants. At room temperature, the ATeam1.03YEMK sensor has a high affinity for ATP with a K_D_^app^ of 0.2 mM compared to cytosolic levels of ATP at 2–10 mM. The ATeamYEMK(R115E) mutant has a lower affinity, similar to wildtype ATeam1.03, with a K_D_^app^ of 0.7 mM. We therefore hypothesized that the R115E mutant would be more sensitive to ATP depletion caused by metabolic inhibitors.

**Figure 6:**
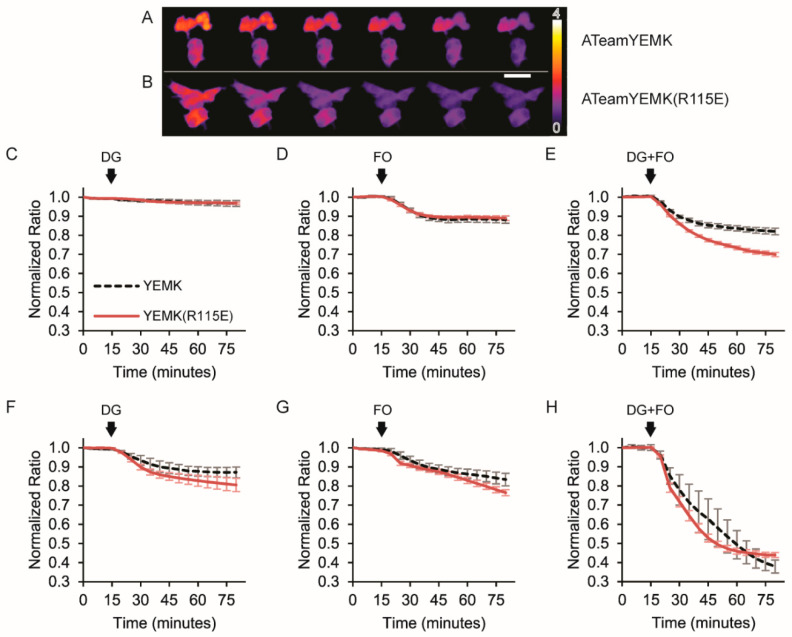
Live-cell imaging illustrates the differential sensitivity of wildtype ATeam1.03YEMK and the R115E lower-affinity mutant. Sensitized FRET emission ratio images of (**A**) wildtype ATeam1.03YEMK and (**B**) the R115E mutant, pseudo-colored to the ratio. Responses to 2-deoxyglucose (DG), FCCP plus oligomycin (FO), or both (DG+FO) (**C**–**E**) in the presence of 10 mM glucose or (**F**–**H**) in the absence of glucose. After baseline measurements for the first 15 min, metabolic inhibitors were added as indicated by the arrow in (**C**–**H**). Inhibitors were maintained in the imaging solution for continuous exposure until the end of the experiment. As expected, responses to metabolic inhibitors in the absence of glucose (**F**–**H**) are more drastic than when glucose is present (**C**–**E**), and the R115E mutant shows increased sensitivity compared to the wildtype.

To test this hypothesis, we first equilibrated cells with 10 mM glucose as well as the mitochondrial substrates glutamine and pyruvate, and then we treated cells with metabolic inhibitors (Figure 6A–E). Metabolic inhibitors were added after a baseline measurement, and inhibitors were continuously maintained in the imaging solution for the remainder of the duration of each experiment. Baseline-normalized responses across cells were highly consistent within each treatment group. The mildest stress was induced by the addition of DG, and it did not cause a drastic loss of ATP as neither the wildtype ATeam1.03YEMK nor the R115E mutant exhibited a change in the sensitized FRET emission ratio (Figure 6C). The addition of FCCP and oligomycin caused a small decrease in the ratio for both sensors that quickly leveled off (Figure 6D). This observation suggests that mitochondrial ATP production is responsible for the bulk of cytosolic ATP, as expected, but glycolytic ATP does contribute and is able to sustain low ATP levels even with mitochondrial impairment. Interestingly, when DG, FCCP, and oligomycin were added together, there was a larger decrease in the R115E mutant signal compared to the wildtype, confirming that the lower-affinity R115E mutant is more sensitive to moderate metabolic stress when glucose is present (Figure 6E).

To investigate responses to more severe metabolic stress, we next treated cells by adding metabolic inhibitors in the absence of glucose (Figure 6F–H). In this case, the addition of DG did cause ATP depletion, and the R115E mutant was more sensitive to reporting this stress as its ratiometric signal decreased to a greater extent than the wildtype ATeam1.03YEMK sensor (Figure 6F). In addition, the R115E mutant responded more quickly to treatments compared to the wildtype for all three combinations of inhibitors (Figure 6F–H).

**Figure 7:**
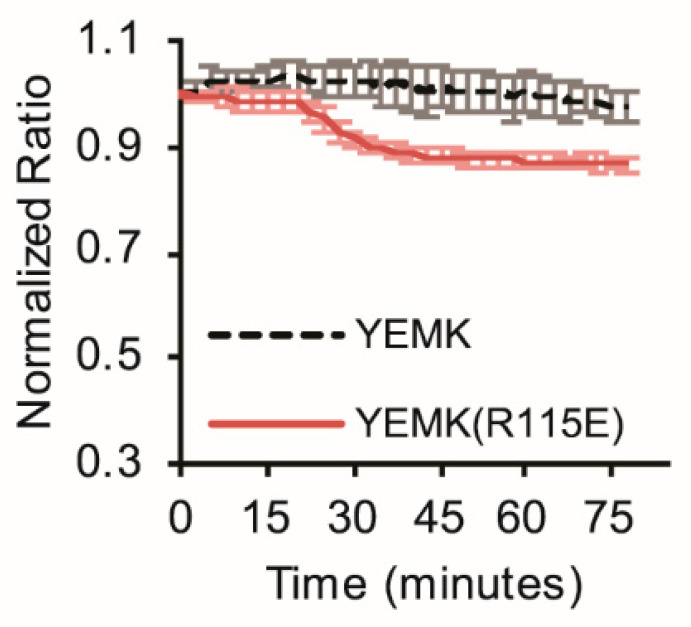
The lower-affinity ATeam1.03YEMK(R115E) mutant is sensitive to mild metabolic stress that is not detected by the wildtype sensor. Cells were imaged in the presence of 2 mM glucose, and 10 mM DG was added at 15 min and maintained in the imaging solution for continuous exposure until the end of the experiment.

Comparison of these experiments illustrates how the presence of glucose poises the cells for their responses to metabolic inhibition. Therefore, as a last demonstration of the differential sensitivity of the sensors, we treated cells with DG in the presence of a lower concentration of 2 mM glucose. With this mild metabolic stress, the wildtype ATeamYEMK did not show a response (Figure 7). In contrast, the R115E reported a clear decrease in cytosolic ATP. Thus, the appropriate choice of an affinity mutant can reveal dynamics that may be obscured otherwise.

## 4. Conclusions

We demonstrated that ATP affinity can be tuned by mutagenesis of the conserved R103 and R115 residues in the ε-subunit ATP-binding domain of the ATeam sensors. Previously, residues 9, 78, 95, and 119 were mutagenized in the *B.* sp. PS3 ε subunit to generate affinity variants optimized for use in mammalian systems at 37 °C [13,14]. To optimize affinity variants for use at a lower room temperature, residues 60 and 132 were also previously explored [8]. However, residues 103 and 115 were not previously interrogated, and here we showed that combinations of charge neutralization and charge reversal mutants have affinities that span 5 orders of magnitude from 200 nM to 2.2 mM.

Interestingly, we found that the structure–function relationship is complex for mutants of ATeam3.10 versus ATeam1.03 and ATeam1.03YEMK despite the high structural homology of the *B. subtilis* and *B.* sp. PS3 ATP-binding ε subunits. Previously, Krah and co-workers hypothesized that the R103A and R115A single mutants would result in increased ATP affinity based on molecular dynamics simulations [18,19]; however, we did not observe a significant decrease in the apparent dissociation constant for these ATeam3.10 mutants. Rather, the R115E, R103A/R115A, and R103A/R115E single and double mutations of the *B.* sp. PS3 ε subunit result in increased ATP and ADP affinity to the ATeam3.10 sensor, although all mutants still exhibited high selectivity for ATP over ADP with 50- to 150-fold difference in ATP versus ADP affinities. The fact that the R-to-E charge reversal mutations also result in increased ATP affinity indicates that residues 103 and 115 have an important secondary influence on the electrostatics of the ATP binding site, but they likely do not directly interact with the phosphates of the nucleotide. In contrast, all mutations caused complete loss of ATP and ADP binding to the ATeam1.03 sensor, and loss of or greatly reduced ATP and ADP binding to the ATeam1.03YEMK sensor. Beyond contributing to our structure–function understanding of the ε ATPase regulatory subunits, these affinity variants also expand the ATP-sensing range at room temperature.

Importantly, we demonstrated with live cell imaging that mutants have differential sensitivity to metabolic stress, and it is critical to select a mutant with an affinity matched to the physiological scenario in order to reveal dynamics that might otherwise be obscured by the insensitivity of an improperly chosen sensor. In the future, it will be interesting to mutagenize these residues together with other key sites to further expand the toolbox of sensors available to study ATP fluctuations in and around cells. In particular, Imamura and co-workers also hypothesized in their original work that exchanging different ε subunits from different species could be a route to generate even more affinity variants [1]. For example, this has been carried out with calcium sensors such as the Twitch series [27]. Interestingly, this has not yet been reported with the ATeam family of sensors and, thus far, affinity mutants have been made using the original *B. subtilis* and *B.* sp. PS3 ε subunits engineered by Imamura and co-workers [1]. This may be in part due to the likely requirement for re-engineering and optimization when exchanging the ATP-binding domain. In addition, a number of ε subunits from different species that have been characterized, and not all bind to ATP within what is thought to be a biologically relevant concentration range, if at all [20,28]. Regardless, in the future, it will also be interesting to more thoroughly investigate the exchange of ATP-binding ε subunits from different species to generate affinity variants across concentration ranges for use at different temperatures.

## Figures and Tables

**Table 1:** Apparent ATP affinities of ATeam3.10 Mutants.

ATeam3.10 Mutation	ATP Affinity K_app_ ^1^
WT	800 ± 300 (200) nM
R103A	800 ± 300 (200) nM
R103E	600 ± 300 (200) nM
R115A	600 ± 200 (200) nM
R115E	300 ± 100 (60) nM *
R103A/R115A	200 ± 100 (90) nM *
R103A/R115E	200 ± 100 (70) nM *
R103E/R115A	700 ± 300 (200) nM
R103E/R115E	800 ± 400 (200) nM

^1^ mean ± standard deviation (95% confidence interval); * significantly different from wildtype (*p* < 0.01, 2-tail *t*-test).

**Table 2:** Apparent ADP affinities of ATeam3.10 Mutants.

ATeam3.10 Mutation	ADP Affinity K_app_
WT	130 ± 10 µM
R103A	82 ± 6 µM
R103E	68 ± 3 µM
R115A	74 ± 7 µM
R115E	29 ± 2 µM
R103A/R115A	32 ± 5 µM
R103A/R115E	17 ± 1 µM
R103E/R115A	40 ± 2 µM
R103E/R115E	51 ± 6 µM

**Table 3:** Apparent ATP Affinity of ATeam1.03 and ATeam1.03YEMK mutants.

ATeam1.03 Mutation	ATP Affinity K_app_ ^1^	ATeam1.03YEMK Mutation	ATP Affinity K_app_ ^1^
WT	1.0 ± 0.5 (0.3) mM	WT	0.2 ± 0.1 (0.09) mM
R103A	Non-Binding	R103A	Non-Binding
R103E	Non-Binding	R103E	ND ^2^
R115A	Non-Binding	R115A	1.8 ± 0.5 (0.5) mM
R115E	Non-Binding	R115E	0.7 ± 0.2 (0.2) mM
R103A/R115A	Non-Binding	R103A/R115A	Non-Binding
R103A/R115E	Non-Binding	R103A/R115E	1.9 ± 0.2 (0.2) mM
R103E/R115A	Non-Binding	R103E/R115A	1.9 ± 0.2 (0.2) mM
R103E/R115E	Non-Binding	R103E/R115E	2.2 ± 0.3 (0.3) mM

^1^ mean ± standard deviation (95% confidence interval); ^2^ Not determined due to reproducibly poor expression.

## Data Availability

Data is available upon request. Plasmid constructs are distributed by Addgene.

## Supplementary Materials

The following supporting information can be downloaded at https://www.mdpi.com/article/10.3390/s25196180/s1, Figure S1: Mutant cycle analysis; Figure S2: ATeam1.03 mutant ADP affinity; Figure S3: ATeamYEMK mutant ADP affinity; Figure S4: Mg^2+^ dependence; Figure S5: R115E Mg^2+^ dependence; Figure S6: Lifetime distributions; Figure S7: Acceptor polarization.
