# Supplementary material for: R103 and R115 Affinity Mutants of ATeam ATP Biosensors
Source: Sensors (Basel). 2025 Oct 6;25(19):6180. doi: 10.3390/s25196180 (PMC12527034; doi:10.3390/s25196180)
Supplement: Supplementary file 1 [file sensors-25-06180-s001.zip › sensors-3835622-supplementary.pdf]

R103 and R115 Affinity Mutants of the ATeam ATP Biosensors

Autumn Cholger [1], Jason M. Conley [1], Stephen A. Valentino [1], Elaine Colomb [1], Olivia de Cuba [2], Jacob Kress [2] and Mathew Tantama [2,*]

[1] Department of Chemistry, Purdue University, West Lafayette, IN 47907, USA
[2] Department of Chemistry & Biochemistry Program, Wellesley College, Wellesley, MA 02481, USA
* Correspondence: mt4@wellesley.edu

Supporting Information

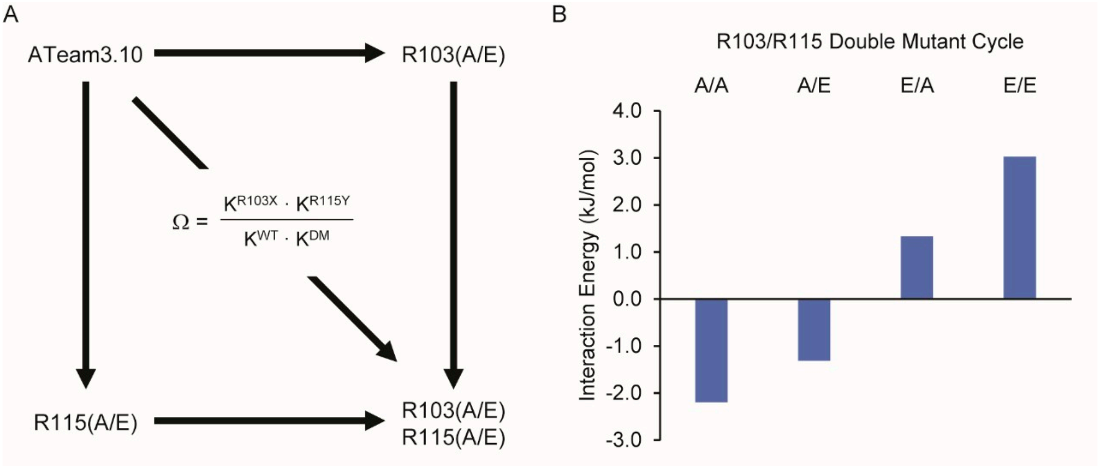

Figure S1. Mutant cycle analysis of ATeam3.10 and its affinity mutants show that there is only weak interaction between positions 103 and 115. (A) Interaction diagram. (B) Interaction energies are weak.

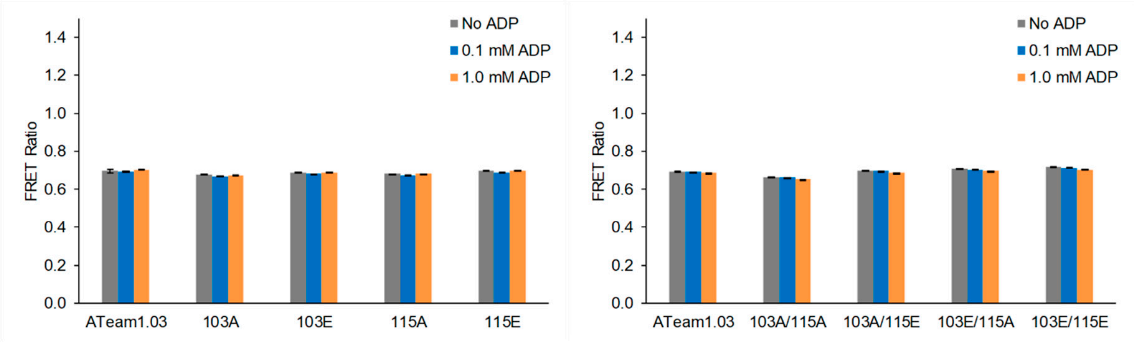

Figure S2. ATeam1.03 R103 and R115 single and double mutants do not bind to ADP. Sensor responses were measured using purified proteins in solution. Average sensitized acceptor emission-to-donor emission FRET ratios are shown, and error bars are 95% confidence intervals. The ATeam1.03 wildtype and all of its mutants showed no significant response to either 0.1 mM (blue) or 1.0 mM (orange) ADP compared to baseline (gray).

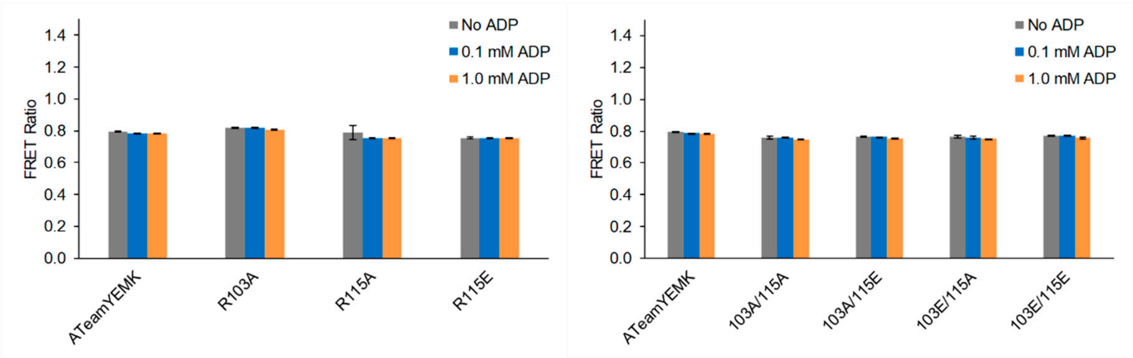

Figure S3. ATeam1.03YEMK ("ATeamYEMK") R103 and R115 single and double mutants do not bind to ADP. Sensor responses were measured using purified proteins in solution. Average sensitized acceptor emission-to-donor emission FRET ratios are shown, and error bars are 95% confidence intervals. The ATeamYEMK wildtype and mutants showed no significant response to either 0.1 mM (blue) or 1.0 mM (orange) ADP compared to baseline (gray).

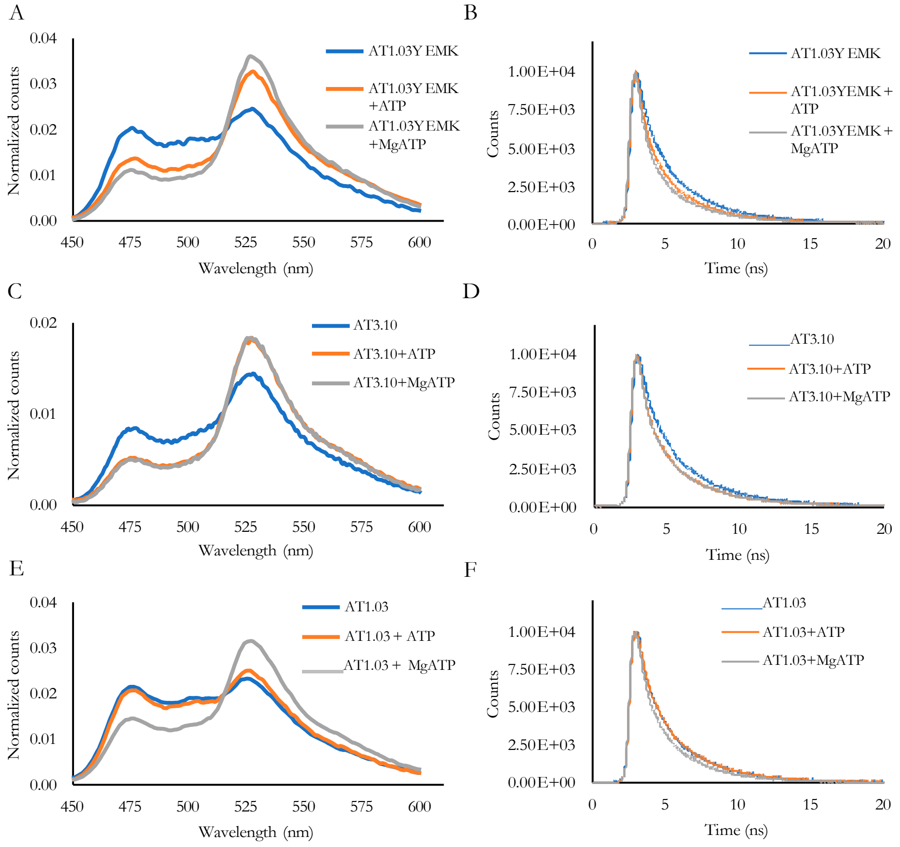

Figure S4. Mg^{2+} dependence for ATP binding to the difference ATeam sensors. Measurements of purified protein in solution. (A,C,E) Emission spectra and (B,D,F) donor lifetime for (A-B) ATeam1.03YEMK, (C-D) ATeam3.10, and (E-F) ATeam1.03. Time-resolved donor lifetime measurements agree with steady-state ratio measurements, validating that the change or lack of change in FRET response to ATP binding is not an artifact of a Mg^{2+} effect on acceptor fluorescence.

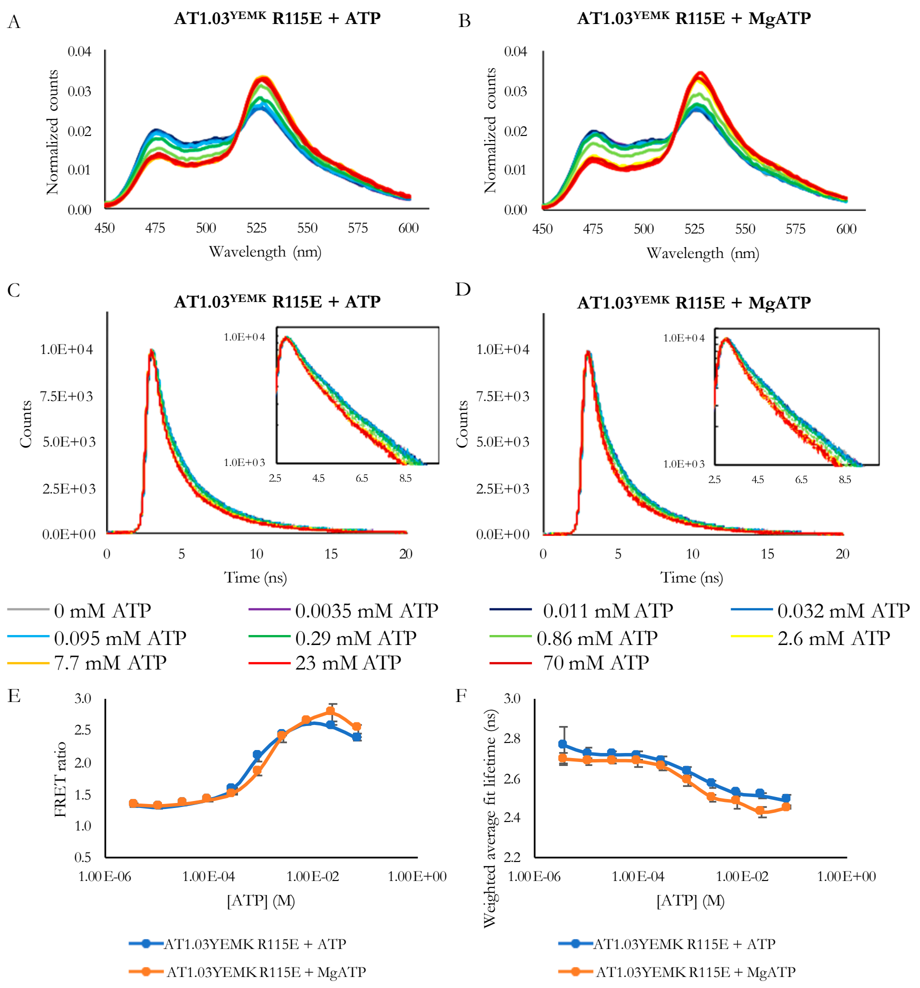

Figure S5. R115E Mg^{2+} dependence. ATeam1.03YEMK(R115E) binds ATP independent of Mg^{2+}. Measurements of purified protein in solution. ATP dose response of the emission spectra in the (A) absence or (B) presence of Mg^{2+}. ATP dose response of donor lifetime decays in the (C) absence or (D) presence of Mg^{2+}. ATP dose response curves for (E) sensitized FRET emission ratio or (F) donor lifetime. Time-resolved donor lifetime measurements agree with steady-state ratio measurements, validating that the change in FRET response to ATP binding is not an artifact of a Mg^{2+} effect on acceptor fluorescence.

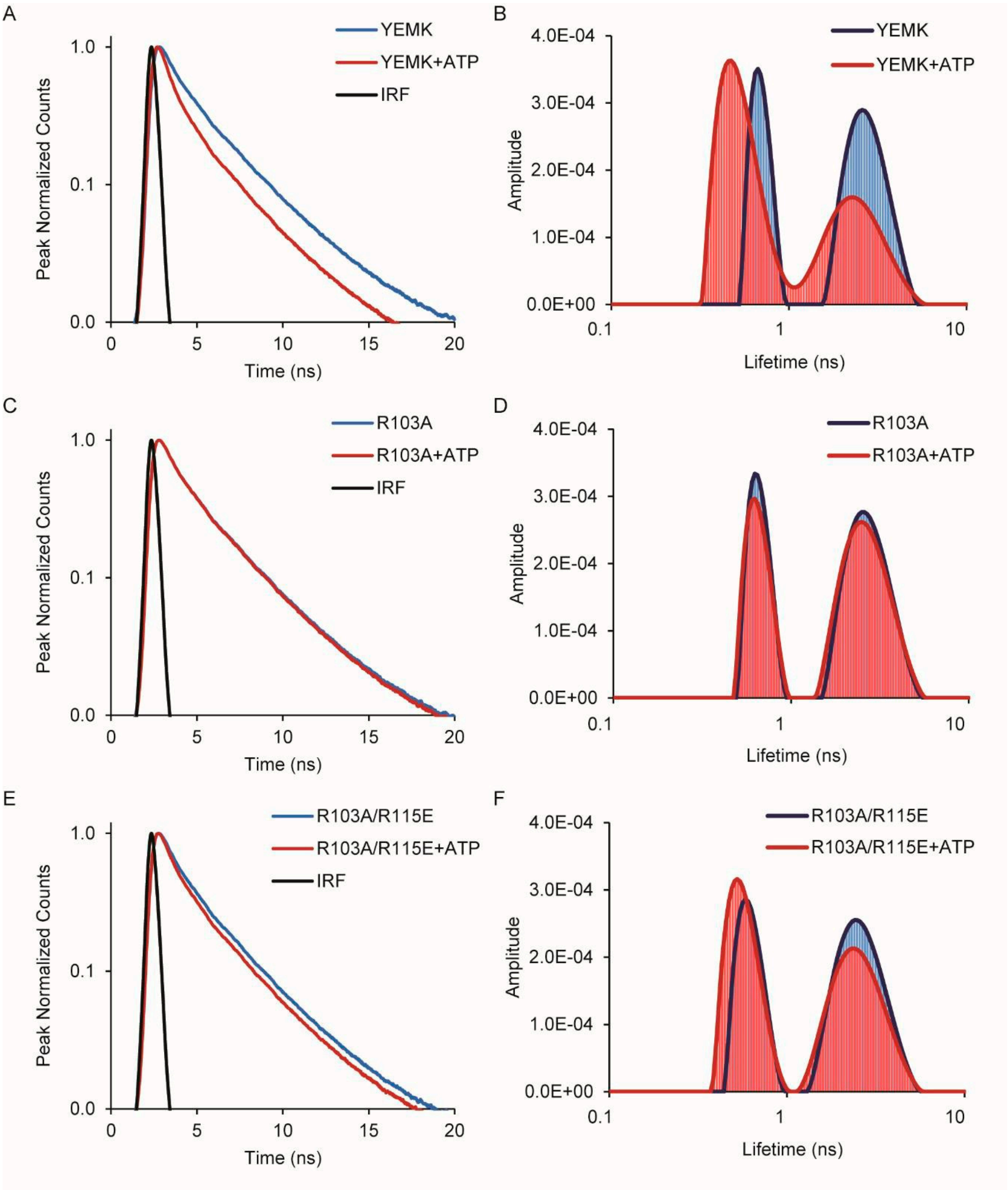

Figure S6. Time-resolved donor lifetime distributions do not provide any evidence of increased conformational dynamics or broadened FRET distributions. Time-resolved mseCFP donor fluorescence lifetime distribution analysis of ATeam1.03YEMK and its mutants measured using purified protein in solution. Representative mseCFP donor fluorescence decays for (A) the wildtype ATeam1.03YEMK, (C) the loss-of-function R103A single mutant, and € the partial-rescue R103A/R115E double mutant. (B, D, F) Lifetime distribution analysis using the maximum entropy method.

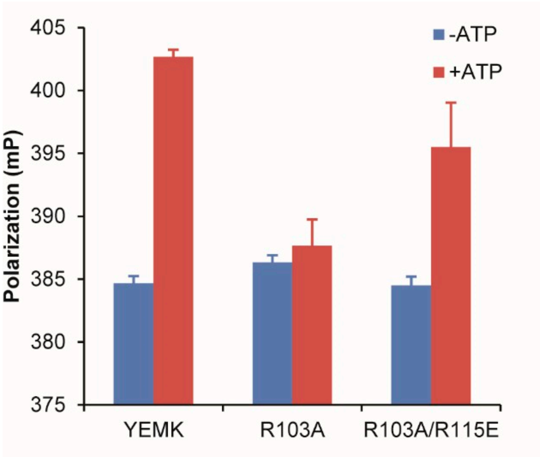

Figure S7. Acceptor fluorescence polarization measurements confirm ATP-induced conformational changes for ATeamYEMK and its R103A/R115E double mutant and lack of conformational change for the R103A single mutant. Steady-state mVenus acceptor fluorescence polarization for ATeam1.03YEMK and its mutants in the absence and presence of ATP. ATP-induced conformational change causes compaction of the ε subunit and decrease in rotation, which increases polarization for ATeamYEMK and the R103A/R115E mutant. Measurements made using purified protein in solution.
